# Supplementary material for: Simulation-based inference of developmental EEG maturation with the spectral graph model
Source: Commun Phys. 2024 Jul 31;7(1):255. doi: 10.1038/s42005-024-01748-w (PMC12310519; doi:10.1038/s42005-024-01748-w)
Supplement: Supplementary file 2 — Supplementary Information [file 42005_2024_1748_MOESM2_ESM.pdf]

Simulation-based Inference of Developmental EEG Maturation with the Spectral Graph Model: Supplementary Information

Danilo Bernardo[1*], Xihe Xie[2], Parul Verma[3], Jonathan Kim[1], Virginia Liu[1], Adam Numis[1], Ye Wu[4], Hannah C. Glass[1,5,6], Pew-Thian Yap[4], Srikantan Nagarajan[3], Ashish Raj[3]

[1*]Department of Neurology, University of California, San Francisco, San Francisco, CA, USA.
[2]Department of Neuroscience, Weill Cornell Medicine, New York, NY, USA.
[3]Department of Radiology, University of California, San Francisco, San Francisco, CA, USA.
[4]Department of Radiology and Biomedical Research Imaging Center, University of North Carolina, Chapel Hill, NC, USA.
[5]Department of Pediatrics, University of California, San Francisco, San Francisco, CA, USA.
[6]Department of Epidemiology and Biostatistics, University of California, San Francisco, San Francisco, CA, USA.

*Corresponding author(s). E-mail(s): dbernardoj@gmail.com;

1 Supplementary Note 1

1.1 Parameter recovery, sensitivity analyses, and simulation based calibration analyses.

In order to assess the accuracy and robustness of the SBI-SGM inference framework, we conducted parameter recovery analyses and simulation-based calibration (SBC) with synthetic data. In the context of SBI, the efficacy of the inference may be evaluated by parameter recovery, specifically—given known ground truth parameters θ, how well does the inference procedure recover them? Running SBI on 100 synthetic

SGM realizations within physiologically informed prior bounds (Table 1), we more accurately recovered SGM parameters with the NPE and TSNPE, relative to NRE (Supplementary Figure 4-7). α was the most accurately recovered parameter across all model families, whereas S and τ_G had the poorest recovery. For parameter recovery across all SGM parameters, NPE had the lowest mean relative estimated error (REE), 0.0451, compared to 0.0560 for TSNPE and 0.114 for NRE (Supplementary Figure 7). To evaluate potential pathologies of the SBI process, we performed Bayesian sensitivity analysis evaluating posterior z-score and posterior contraction (Supplementary Figure 8) for each parameter using NRE, NPE, and TSNPE across various simulation budget sizes. We demonstrate that relative to NPE and NRE, TSNPE provided improved posterior contraction (Supplementary Figures 9 and 10). Systematic posterior underdispersion or overdispersion limited usefulness of posterior z-scores in comparing different model families and their respective parameterizations, particularly for NRE, whose posteriors demonstrated increased posterior dispersion relative to NPE and TSNPE. We evaluated posterior dispersion indices (PDI), which quantify the degree of posterior dispersion, standardizing for the varied scale of different SGM parameters. We demonstrate that NPE and TSNPE had reduced PDI relative to NRE (Supplementary Figure 11).

To evaluate the calibration of the uncertainties of the estimated posteriors, we assessed the SBC of NPE and TSNPE. NRE was excluded from SBC analysis because the NRE-based inference of SGM parameters proved computationally prohibitive due to the utilization of MCMC sampling. We found that the TSNPE yielded posteriors with well-calibrated uncertainties, whereas NPE yielded posteriors with left-skewed rank distribution consistent with systematic underestimation of the posterior means (Supplementary Figure 12). To quantify SBC, we utilized the Kolmogorov-Smirnov (KS) test to evaluate the null hypothesis that the samples from ranks are drawn from a uniform distribution. TSNPE SBC KS testing demonstrated p-values over 0.05 for all SGM parameters, which suggests the posterior distribution aligns with a uniform distribution, aligning with a necessary, though not sufficient, condition for the estimated posterior to be accurately calibrated. In contrast, KS testing for NPE across all SGM parameters demonstrated $p < 0.001$. In addition, we performed the Classifier 2-Sample Test (C2ST) to assess whether the estimated posterior is drawn from the same distribution as the prior, and this demonstrated that the Data Averaged Posterior (DAP) compared to the prior demonstrated C2ST values between 0.473 to 0.510, approximating 0.5, indicating statistical similarity of the DAP distribution to the prior distribution. The performance differences between NPE and TSNPE are characterized by superior calibration in TSNPE and lower relative estimation error in parameter recovery for NPE. This distinction highlights that greater accuracy in parameter estimates does not necessarily correspond to an accurate representation of true data uncertainties.

2 Supplementary Note 2

2.1 Performance differences between NRE, NPE, and TSNPE

Here, we evaluate potential sources of the differential performance observed with NRE, NPE, and TSNPE. In evaluating potential sources of error in parameter recovery and calibration, we observed a tendency for NPE to exhibit posterior mass leakage beyond the defined prior boundaries leading to clustering of posterior mass at prior boundaries, as previously reported by Deister et al[1]. NPE showed clustering of posterior mass near the boundary limits for parameters, especially with conduction speed. In contrast, TSNPE demonstrated no such leakage (Supplementary Figure 13). Despite the potential leakage, the estimated posterior within the prior bounds for NPE was accurate in capturing true parameters as evidenced by its higher REE scores; consistent with prior findings by Diestler et al. that despite leakage, the approximate posterior within the bounds of the prior may still be reflective of the true posterior[1]. However, this leakage negatively impacts the posterior distribution shape estimated by NPE, particularly impacting parameters with complex identifiability, such as conduction speed. This issue may contribute to the poorer calibration results observed with NPE compared to TSNPE. While transformations of parameter space and other remedies for NPE leakage have been proposed, Diestler et al. noted that these modifications to NPE did not sufficiently rectify the shortcomings in real-world data, whereas TSNPE effectively mitigated leakage[1]. Our findings indicate improved posterior calibration with TSNPE, aligning with the findings of Deistler et al. that TSNPE may recover posteriors with good calibration more effectively than NPE[1].

Next, upon inspection of posterior predictive checks (PPC) utilizing synthetic spectral realizations, we observed that the estimated posterior distributions qualitatively demonstrated interdependencies among certain SGM parameters, which varied according to SBI method utilized (Supplementary Figures 4-6). We assessed these correlations using the Pearson correlation coefficient matrix for the joint marginal distributions (Supplementary Figure 14). There were strong correlations across several joint marginals of the estimated posterior distribution, indicative of model degeneracy, wherein multiple model parameterizations yield similar spectral realizations. NPE had increased sensitivity to detect correlations between parameters compared to TSNPE. Increasing simulation budget size with TSNPE led to increased alignment in detected significant correlations with NPE, suggesting that increased simulation budget size for neural density estimator training increases its ability to capture interdependencies in the multivariate posterior distribution. However, when averaging across the entire synthetic dataset, no significant correlations were observed. This observation suggests that while model degeneracy may explain the correlations seen in individual joint marginal distributions, it may not manifest uniformly against the variance representative of spectral developmental trajectories.

Concerning the improved REE performance of NPE compared to TSNPE, we attribute this to the larger simulation budgets allocated to NPE relative to TSNPE due to the computational efficiency of amortized inference of NPE compared to the non-amortized inference of TSNPE. Larger simulation budgets generally lead to performance gains for NPE-based methods [2]. However, for TSNPE, this comes at the

expense of computational complexity that scales linearly with the number of subjects because a new neural density estimator is learned for each subject at each inference round for TSNPE. In comparison, with NPE, a single trained neural density estimator is re-used across all subjects.

3 Supplementary Figures

Supplementary Figure 1: Empirical Subject EEG Spectra

a

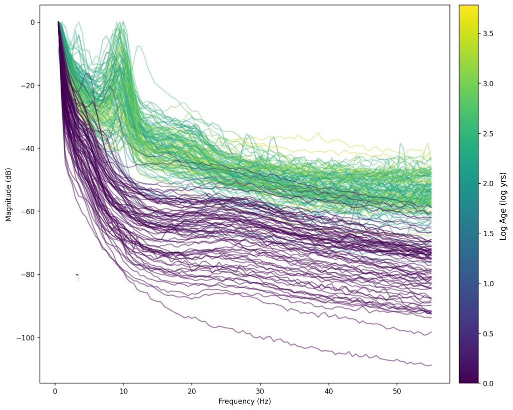

b

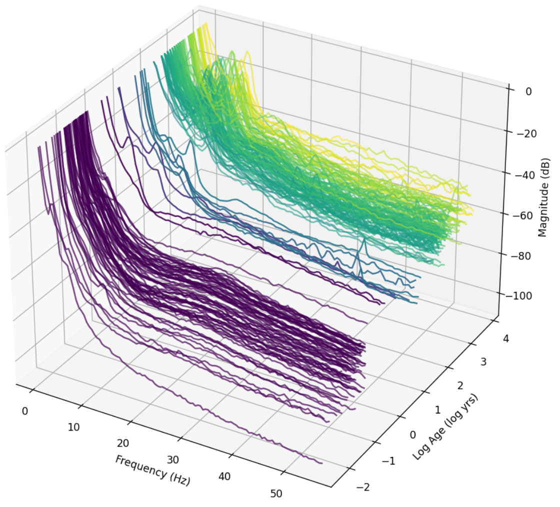

a) demonstrates all subject spectra (whole brain averaged) colored by age, color-mapped to log years. For visualization purposes, all spectra are scaled to the power corresponding to the 0.5 to 1 Hz frequency bin. In this 3-dimensional representation, age is mapped to the z-axis in a plane perpendicular to the page. b) demonstrates all subject spectra colored by age (whole brain averaged), color-mapped to log years. For visualization purposes, all spectra are scaled to the power corresponding to the 0.5 to 1 Hz frequency bin.

Supplementary Figure 2: UMAP of Subjects and Simulated EEGs

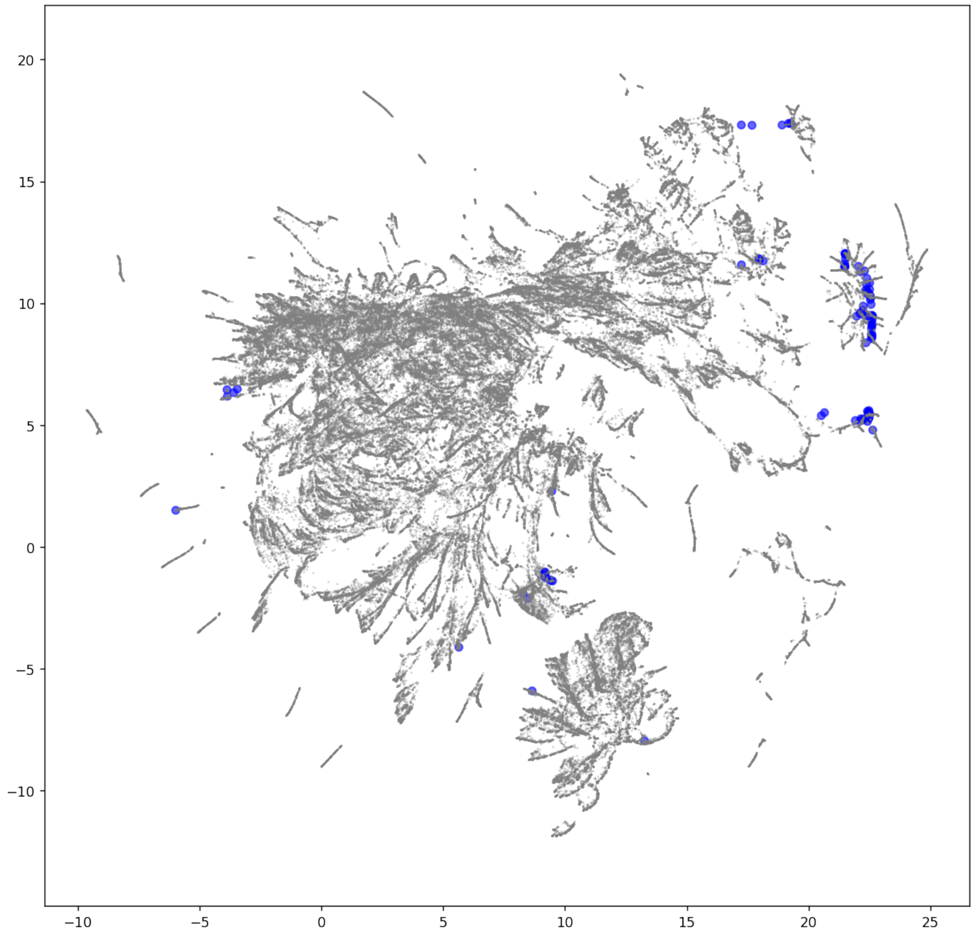

Uniform Manifold Approximation and Projection (UMAP) was used to generate low dimensional embeddings of simulated SGM spectra and observed subject EEG spectra to evaluate their similarity. The UMAP embedding (N=196000 simulations) demonstrates overlap between simulation EEG spectra embeddings and observed EEG spectra embeddings, with with simulations colored in grey and subjects colored in blue. This indicates that SGM parameter variation replicates real-world EEG spectral features observed across development.

Supplementary Figure 3: Effects of Neonatal versus Adult Connectome and Weak Versus Strong Long-range Coupling on SGM Spectra

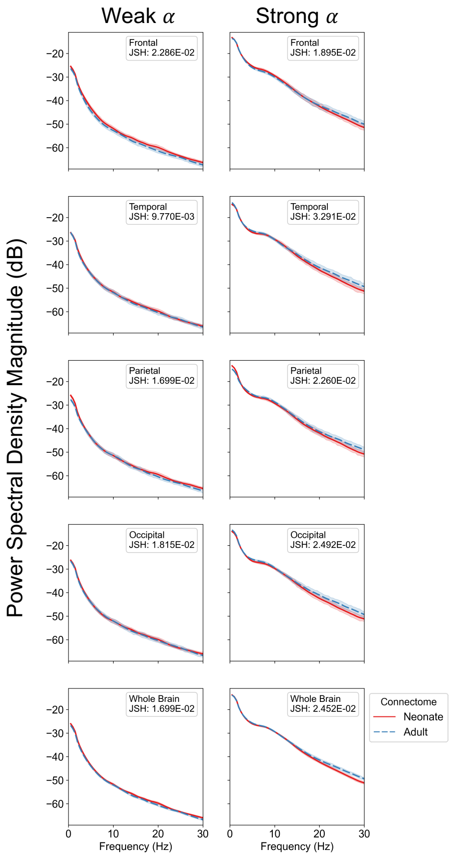

We compared the differential effects of utilizing a neonate versus adult connectome and strong versus weak long-range coupling (α) on Spectral Graph Model (SGM) power spectral density (PSD) realizations across different brain regions (frontal, temporal, parietal, occipital, and whole-brain). Here, we align SGM spectral realizations in each column by weak or strong α regime (as opposed to connectome alignment shown in Figure 3) to more directly assess change in PSD distribution induced by connectome within each subplot. The left and right columns demonstrate SGM PSD realizations instantiated with weak or strong α. Each subplot shows mean SGM PSD realizations per brain region instantiated with neonate (red line) and adult (blue-dotted line) group-averaged connectomes (N = 1000 per connectome), with 95% confidence intervals (CI) indicated by the corresponding shaded regions. α was sampled uniformly at random between 0.1 to 0.3 for weak and between 0.7 to 0.9 for strong α regimes, respectively. The remaining SGM parameters were sampled uniformly at random from physiologically informed prior ranges (Table 1). There were subtle region-specific PSD distribution differences between adults and neonates, reflecting variations in anatomical structural connectivity. Jensen-Shannon divergences induced by connectome selection were relatively smaller compared to those induced by α selection.

Supplementary Figure 4: Parameter Recovery Analyses with Neural Ratio Estimation of SGM Parameters with Synthetic Spectra

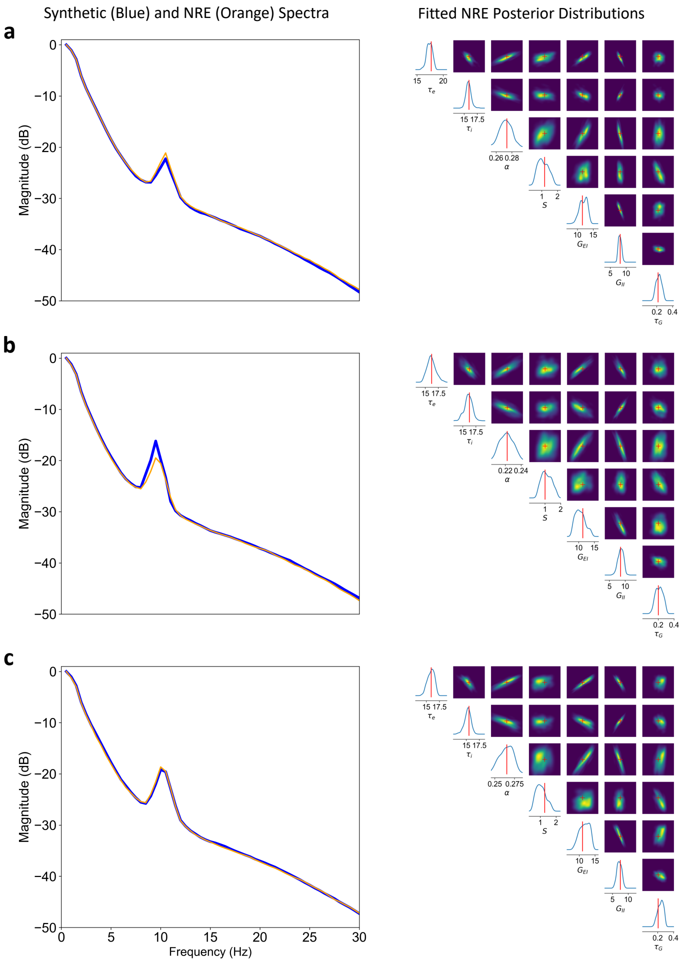

a-c) demonstrate examples of synthetic EEG spectra (left panels, blue traces), corresponding Neural Ratio Estimation (NRE) of Spectral Graph Model (SGM) parameter posterior distributions (right panels), and corresponding inferred SGM Simulated spectra (left panels, orange traces) demonstrating the accuracy of parameter recovery. NRE with a simulation budget size of 1E6 simulations was used to infer SGM parameter posterior likelihoods from synthetic subjects (three examples shown in **a-c**). The left column shows three examples (a-c) of synthetic (blue) and fitted EEG spectra (orange). Kernel density estimation (KDE) of the resulting estimated posterior probability distribution and the true value for each SGM parameter (red cross) are shown in the right column.

Supplementary Figure 5: Parameter Recovery Analyses with Neural Posterior Estimation of SGM Parameters with Synthetic Spectra

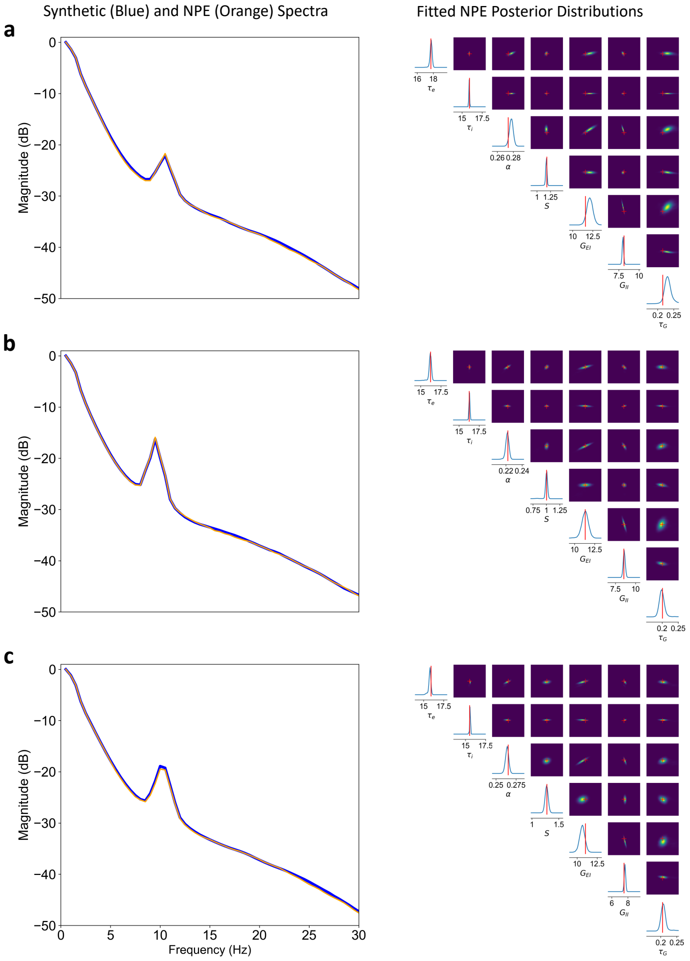

a-c) demonstrate examples of synthetic EEG spectra (left panels, blue traces), corresponding Neural Posterior Estimation (NPE) of Spectral Graph Model (SGM) parameter posterior distributions (right panels), and corresponding inferred SGM Simulated spectra (left panels, orange traces) demonstrating the accuracy of parameter recovery. NPE with simulation budget of 1E6 simulations was used to infer SGM parameter posterior likelihoods from synthetic subjects (three examples shown in **a-c**). The left column shows three examples (a-c) of synthetic (blue) and fitted EEG spectrum (orange). Kernel density estimation (KDE) of the resulting estimated posterior probability distribution and the true value for each SGM parameter (red cross) are shown on the right column.

Supplementary Figure 6: Parameter Recovery Analysis with Truncated Sequential Neural Posterior Estimation of SGM Parameters with Synthetic Spectra

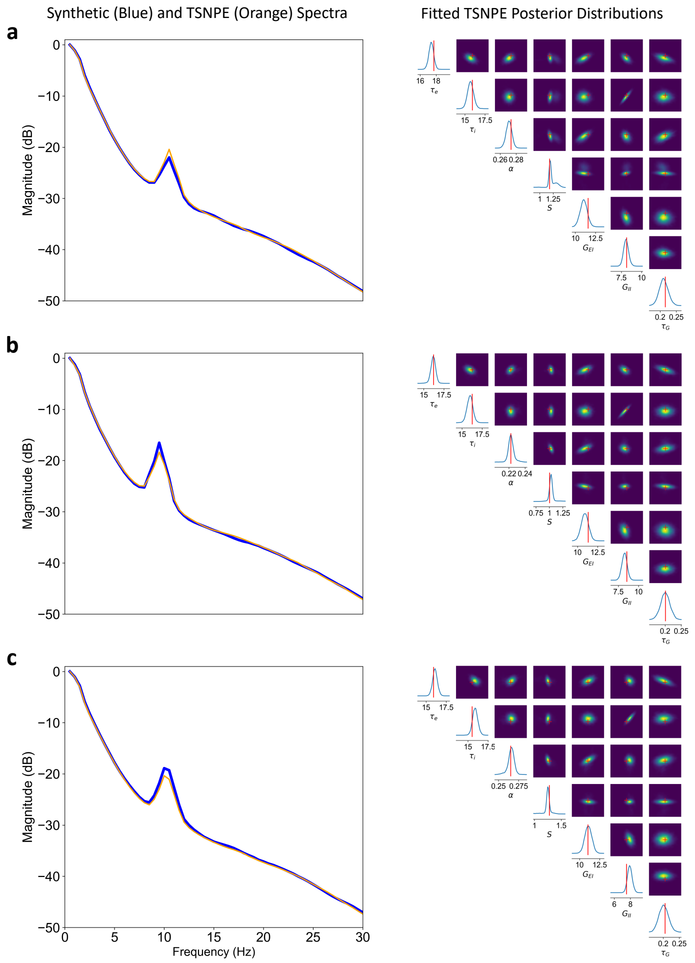

a-c) demonstrate examples of synthetic EEG spectra (left panels, blue traces), corresponding Truncated Sequential Neural Posterior Estimation (TSNPE) of Spectral Graph Model (SGM) parameter posterior distributions (right panels), and corresponding inferred SGM Simulated spectra (left panels, orange traces) demonstrating the accuracy of parameter recovery. TSNPE with three rounds and simulation budget of 2000 simulations was used to infer SGM parameter posterior likelihoods from synthetic subjects (three examples shown in **a-c**). The left column shows three examples (a-c) of synthetic (blue) and fitted EEG spectrum (orange). Kernel density estimation (KDE) of the resulting estimated posterior probability distribution and the true value for each SGM parameter (red cross) are shown on the right column.

Supplementary Figure 7: Parameter Recovery Analysis

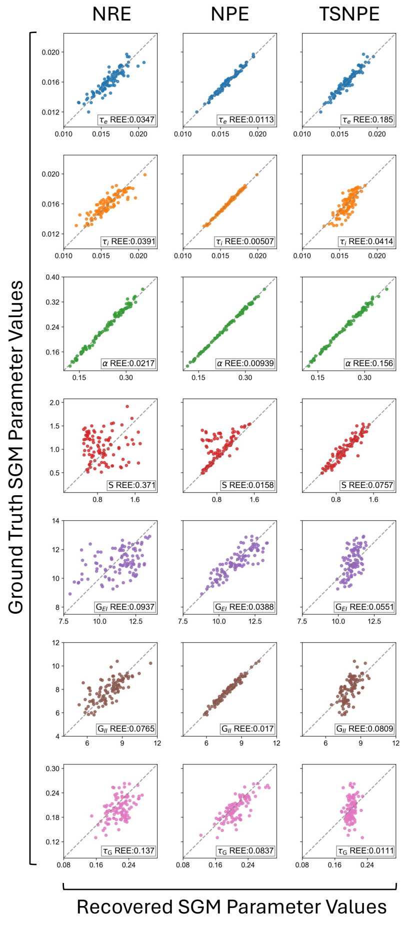

Spectral Graph Model (SGM) parameter recovery performance with simulation-based inference (SBI) using 100 synthetic SGM realizations within physiologically informed prior bounds (Table 1). The predicted values versus the true values of each parameter are shown, respectively, with the gray diagonal line indicating the perfect recovery. The far left column shows parameter recovery with Neural Ratio Estimation (NRE) utilizing 1E6 simulations, the center column demonstrates Neural Posterior Estimation (NPE) utilizing 1E6 simulations, and the right column shows parameter recovery with Truncated Sequential Neural Posterior Estimation (TSNPE) utilizing three rounds and 2000 simulations. α had the best R^2 across all model families, while S, G_{EI}, and τ_G had the poorest recovery error. Across all SGM parameters, NPE overall had improved mean relative estimated error (REE) compared to TSNPE and NRE (0.0451 versus 0.0560 and 0.114, respectively).

Supplementary Figure 8: Bayesian Sensitivity Analysis

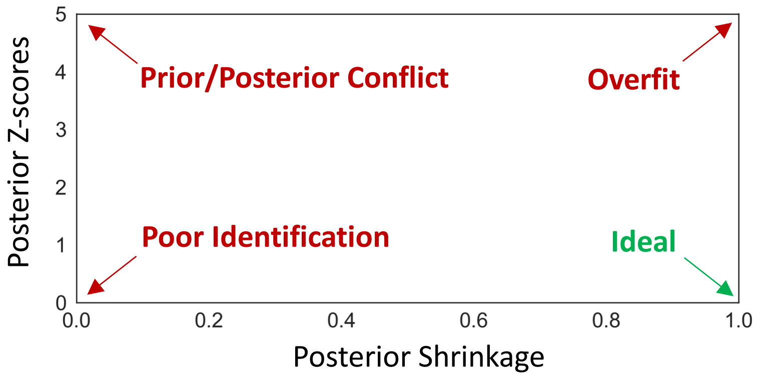

Bayesian sensitivity analyses can be used to identify pathology in the simulation-based inference (SBI) procedure. To assess the inference's reliability with synthetic data, we analyzed the relationship between posterior z-scores and posterior shrinkage. This analysis can identify regions of poor identification, overfitting, and prior/posterior conflict. z-scores were calculated as $\text{Posterior } z\text{-score} = |\mu_n(\tilde{y}) - \theta_n/\sigma_n(\tilde{y})|$ and shrinkage as $\text{Posterior Shrinkage} = 1 - \sigma^2_{\text{prior}}/\sigma^2_{\text{post}}$, following Betancourt et al.[3].

Supplementary Figure 9: Bayesian Sensitivity Analysis of Simulation-based Inference of SGM Parameters

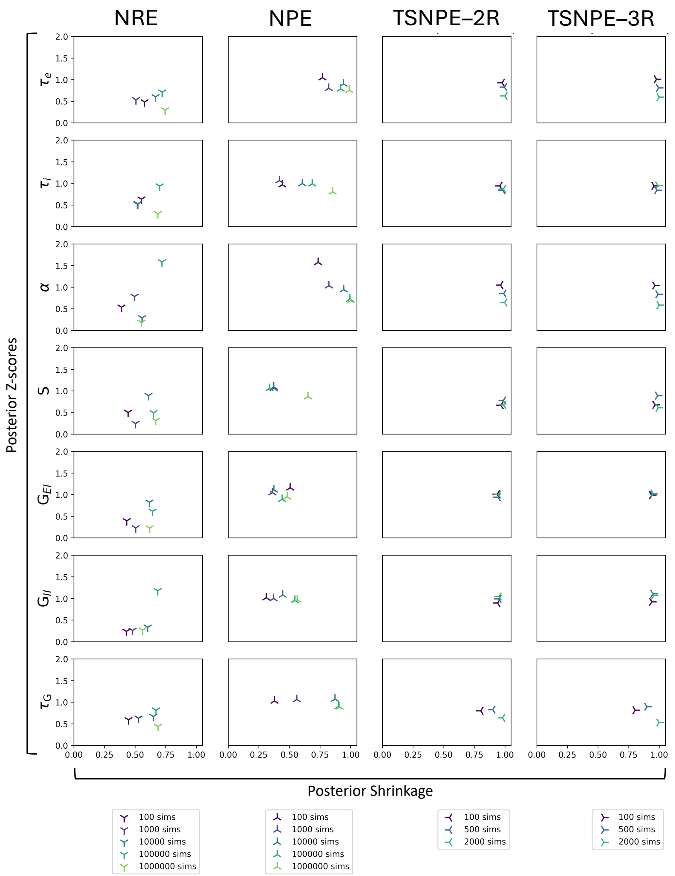

Bayesian sensitivity analyses were used to identify pathology in the simulation-based inference (SBI) procedure. We compared Neural Posterior Estimation (NPE) across varying simulation budget sizes (100 to 1E6 simulations) to Truncated Sequential Neural Posterior Estimation (TSNPE) at varying simulation budget sizes (100 to 2000) and varying number of rounds (two or three). Mean posterior shrinkage and z-scores from each of these models are shown for inference from 100 synthetic Spectral Graph Model (SGM) simulations. TSNPE with three rounds and 2000 simulations overall exhibited the largest posterior shrinkage and lowest posterior z-scores. The posterior mean of the specified simulation run is plotted in each subplot. Individual z-scores and posterior shrinkages from all 100 synthetic SGM simulations for TSNPE with three rounds and 2000 simulations are demonstrated in Supplementary Figure 9.

Supplementary Figure 10: Bayesian Sensitivity Analysis - TSNPE with Three Rounds and 2000 Simulations

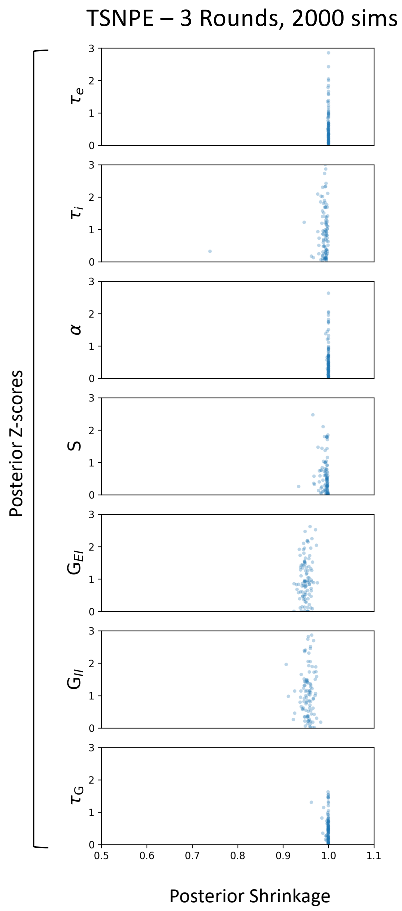

Posterior shrinkages and z-scores are shown for 100 synthetic simulations for each Spectral Graph Model (SGM) parameter utilizing Truncated Sequential Neural Posterior Estimation (TSNPE) with three rounds and 2000 simulations. There was good posterior shrinkage and posterior z-scores with good behavior for most simulations; however, for each parameter there was a small tail of realizations that were overfitted.

Supplementary Figure 11: Posterior Dispersion Indices Analysis

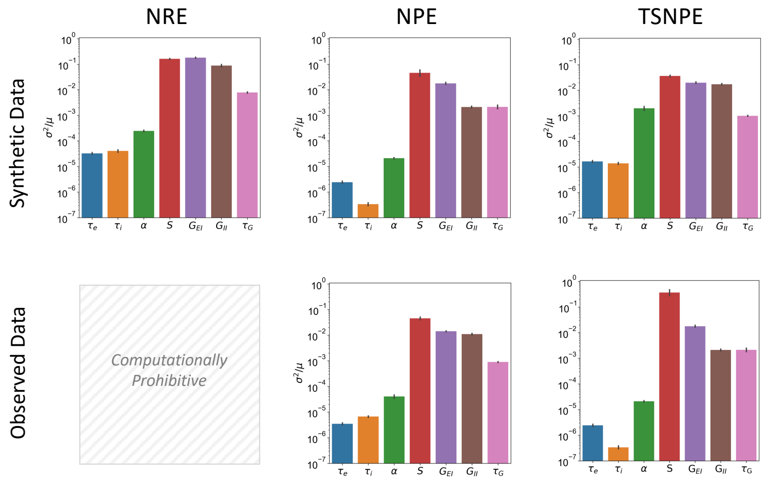

Posterior dispersion indices (PDI) were obtained by normalizing variance by respective parameter mean in order to account for variation in scales across Spectral Graph Model (SGM) parameters. Sequential Neural Posterior Estimation (NPE) and Truncated Sequential Neural Posterior Estimation (TSNPE) had reduced PDI relative to Neural Ratio Estimation (NRE). NPE and TSNPE had similar PDI profiles. PDI across synthetic and observed datasets revealed highest PDI in conduction speed and excitatory:inhibitory gains; aligning posterior predictive check findings that these values had relatively more degeneracy. NPE was performed with 1E6 simulations. TSNPE was performed with three rounds and 2000 simulations. The application of NRE to empirical data was computationally prohibitive due to its utilization of MCMC sampling. Symbols and abbreviations: Excitatory time constant, τ_{e} (blue); Inhibitory time constant, τ_{i} (orange); Long-range coupling constant, α (green); Conduction velocity, S (red); Excitatory gain, G_{EI} (purple); Inhibitory gain, G_{II} (brown); Graph time constant, τ_{G} (pink).

Supplementary Figure 12: Simulation-based Calibration Analysis

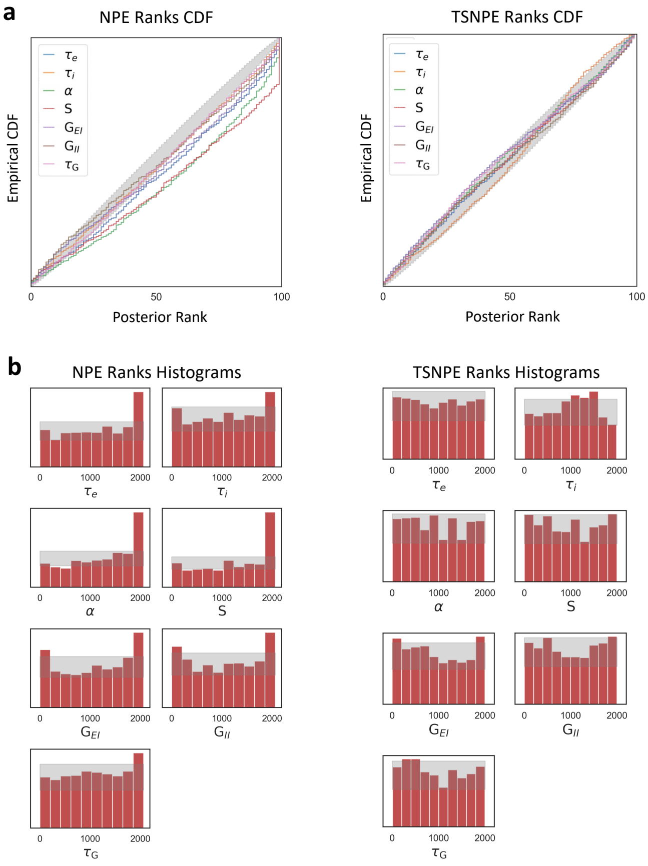

a) The cumulative distribution functions (CDF) of posterior ranks are demonstrated for every parameter (colored lines), compared to the 95% confidence interval of the uniform distribution (gray diagonal region) for both Sequential Neural Posterior Estimation (NPE) and Truncated Sequential Neural Posterior Estimation (TSNPE). NPE demonstrates a ranks CDF below the gray region, indicative of a non-uniform distribution, whereas TSNPE ranks CDF lie within the gray region. b) Inspection of the rank histograms for NPE revealed a right-skewed rank distribution indicative of systematic underestimation of the posterior mean. Each rank histogram demonstrates binned estimated CDF values (red) along the x-axis. Inspection of the ranks histograms for TSNPE revealed relatively uniform distributed ranks across parameters; however, with subtle U-shaped distribution for τ_{i} and α consistent with mild underestimation of the posterior variance. NPE was performed with 1E6 simulations. TSNPE was performed with three rounds and 2000 simulations. Symbols and abbreviations: Excitatory time constant, τ_{e} (blue); Inhibitory time constant, τ_{i} (orange); Long-range coupling constant, α (green); Conduction velocity, S (red); Excitatory gain, G_{EI} (purple); Inhibitory gain, G_{II} (brown); Graph time constant, τ_{G} (pink).

Supplementary Figure 13: Posterior Leakage with Neural Posterior Estimation

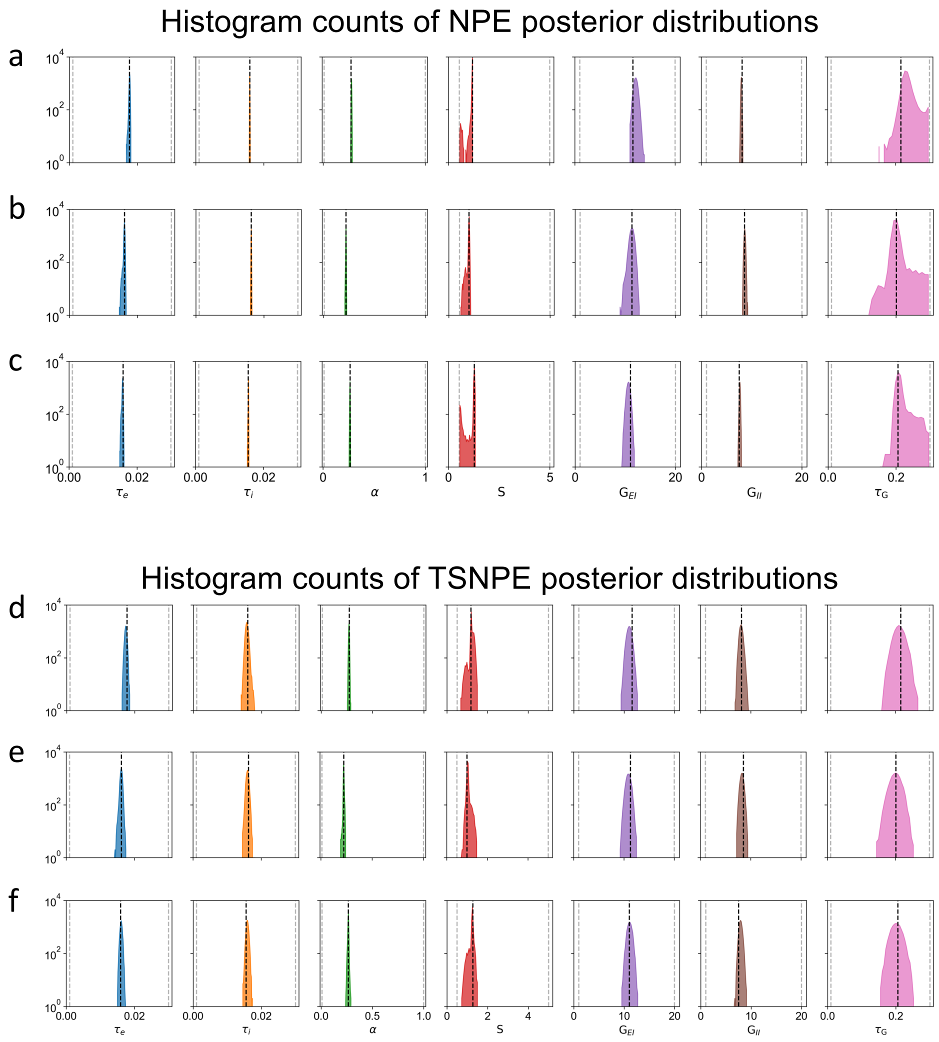

Histogram counts of Neural Posterior Estimation (NPE) and Truncated Sequential Neural Posterior Estimation (TSNPE) estimated posterior distributions are shown, with log-scaling of the y-axis to identify regions containing posterior mass not readily visible in Supplementary Figures 4-6. NPE exhibited clustering of posterior mass at the prior boundary for conduction speed, indicative of posterior leakage[1]. TSNPE did not exhibit any signs of posterior leakage. NPE was performed with 1E6 simulations. TSNPE was performed with three rounds and 2000 simulations.

Supplementary Figure 14: Posterior Distribution Pearson Correlation Matrices

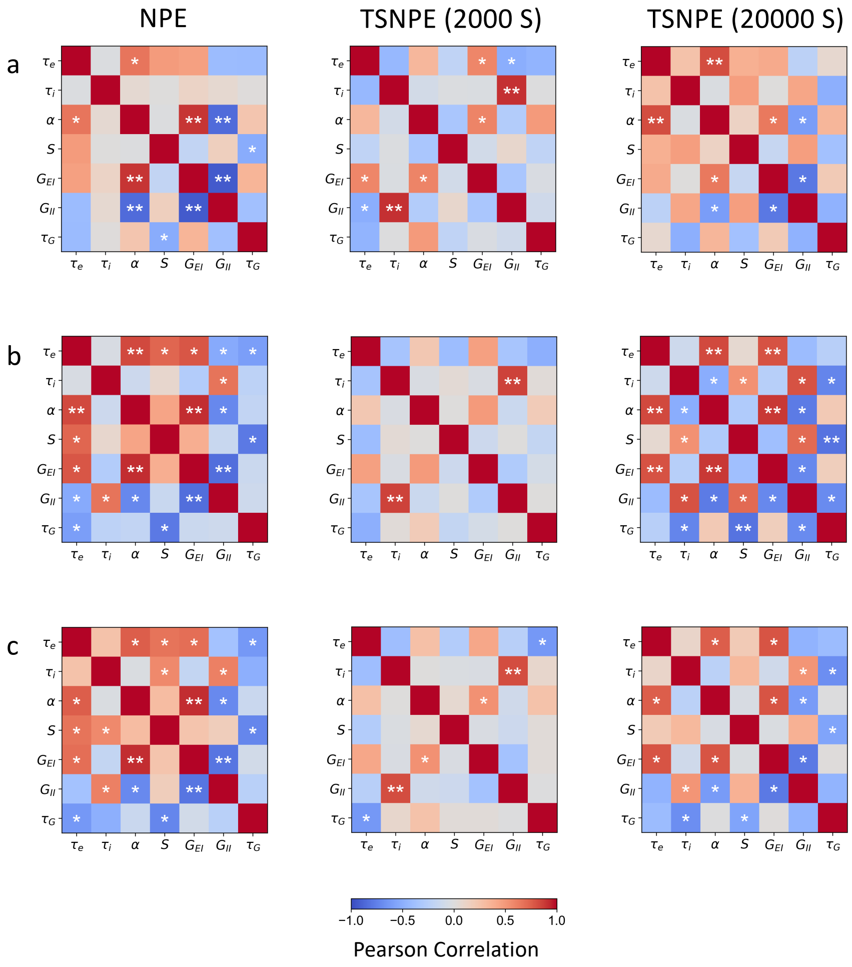

We assessed for correlations in the estimated posterior joint marginal distributions utilizing Pearson correlation coefficient (R^2) matrices. Rows a through c correspond with synthetic data labeled a through c in Supplementary Figures 4-6. There were significant, strong correlations ($R^2 > 0.8$) denoted by ** and significant, moderate correlations ($R^2 > 0.5$) across several joint marginals of the estimated posterior distribution. All denoted significant correlations had $p < 0.001$, adjusted for multiple comparisons using Bonferroni correction. These significant correlations indicate model degeneracy, wherein multiple model parameterizations yield similar spectral realizations. Neural Posterior Estimation (NPE) performed with 1E6 simulations demonstrated increased sensitivity to detect correlations between parameters when compared to Truncated Sequential Neural Posterior Estimation (TSNPE) utilizing two rounds of sequential inference with a simulation budget size of 2000. Increasing the simulation budget size to 20000 simulations with TSNPE led to increased alignment in detected significant correlations with NPE. When averaging across all realizations of the entire synthetic dataset, no significant correlations were observed.

Supplementary Figure 15: Posterior Distribution for All Subjects: Part A

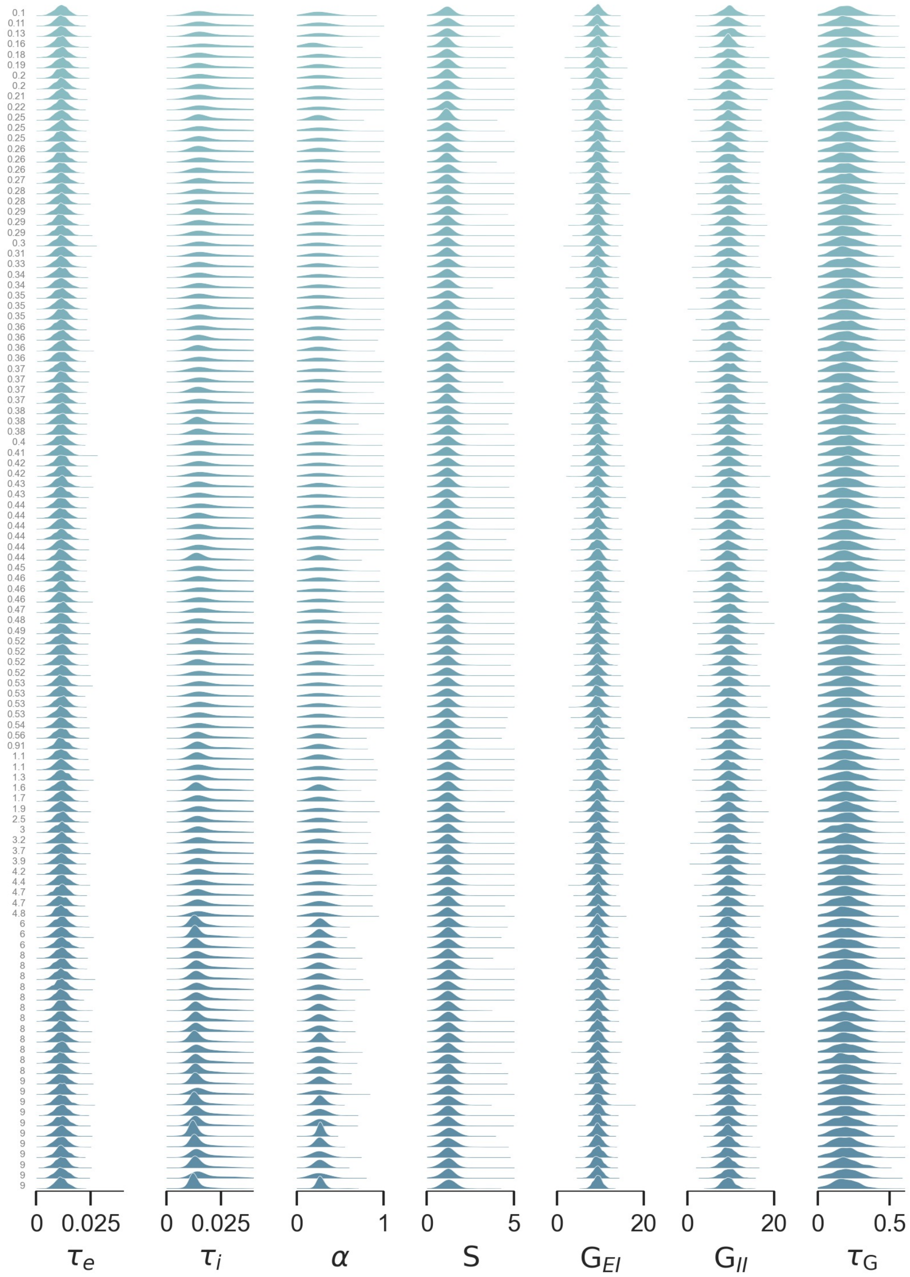

The posterior distribution is shown for all subjects, ordered by age. The remainder of the subjects are shown in Supplementary Figure 16

Supplementary Figure 16: Posterior Distribution for All Subjects: Part B

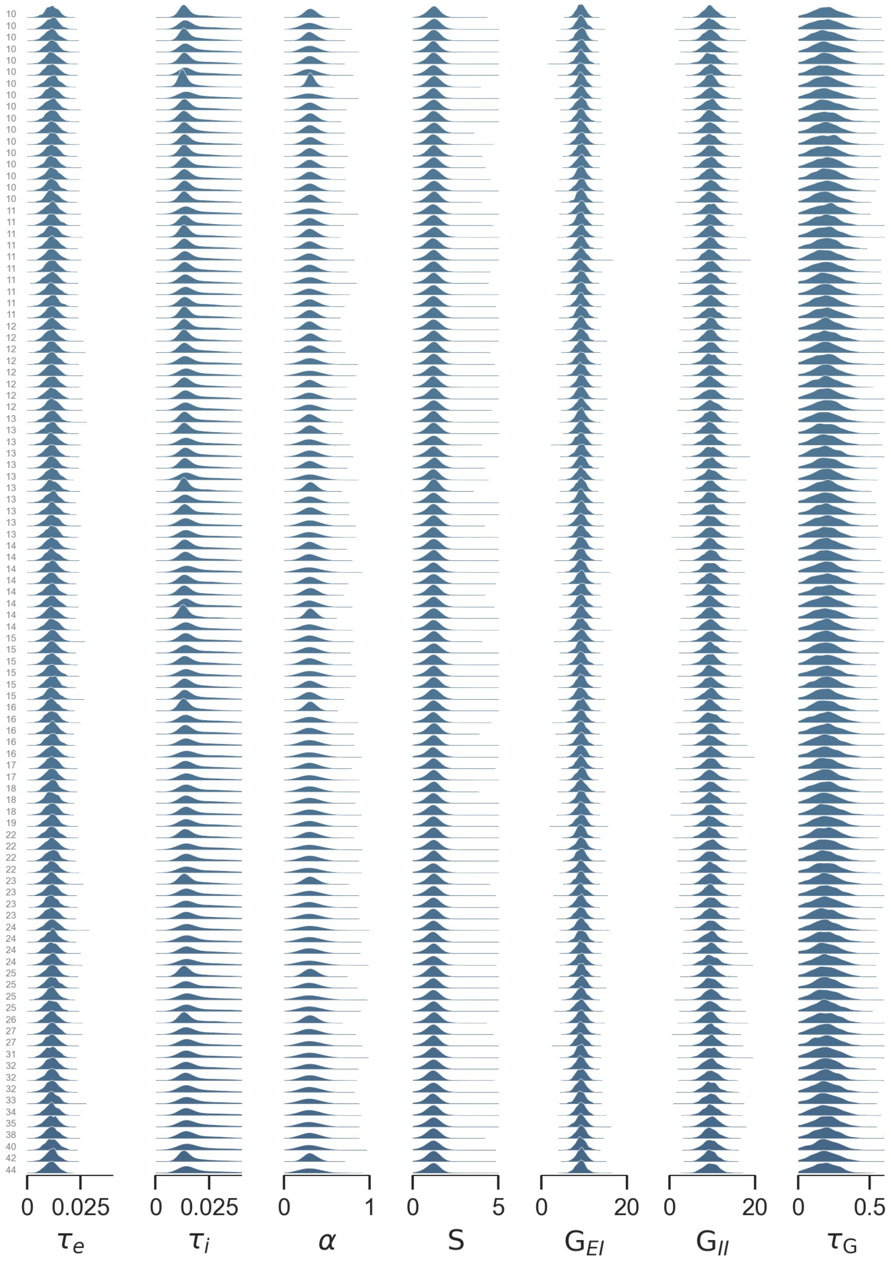

The posterior distribution is shown for all subjects, ordered by age (continuation from Supplementary Figure 15).

Supplementary Figure 17: τ_G Relation with Age

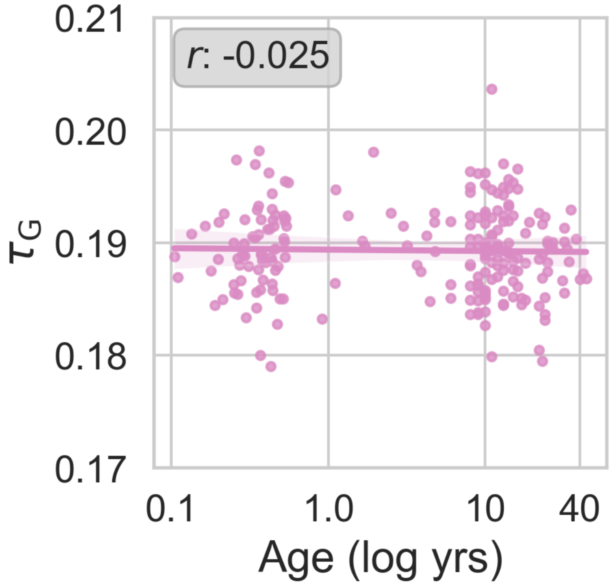

The graph time constant, τ_G, did not demonstrate time-dependent change during development.

Supplementary Figure 18: Regression Diagnostics for SGM Parameter Relation with Age

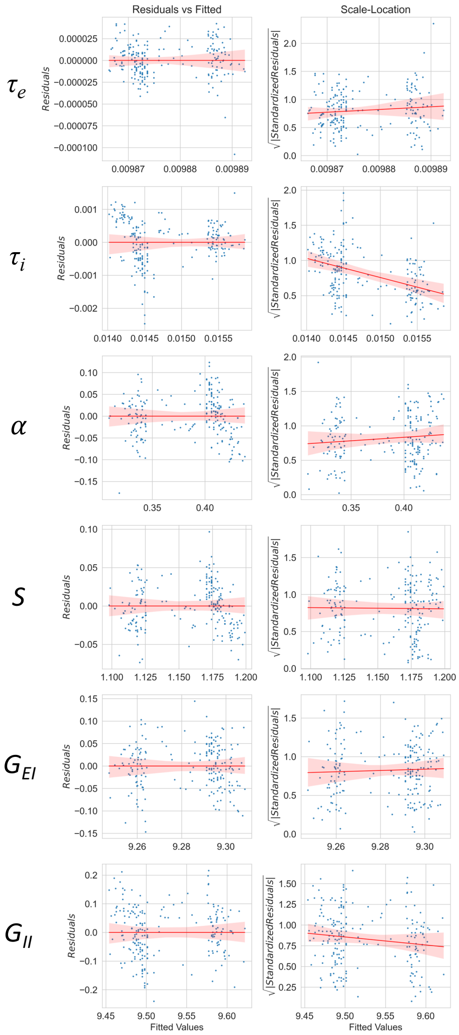

The left column demonstrates residuals vs. fitted values, and the right column demonstrates scale-location plots across the Spectral Graph Model (SGM) parameters found to have a linear correlation with age. The red line in the residuals vs. fitted plots shows the relationship between the fitted values (predicted values) and the residuals for each subject (blue dot), with the shaded regions indicating the 99.9% confidence interval. The red lines in the scale-location plots show the relationship between the fitted values (predicted values) and the square root of the absolute standardized residuals, with shaded regions indicating the 99.9% confidence intervals. Significant heteroskedasticity was found for τ_e and τ_i with BP values of 5.560 and 14.13, with p-values of 0.0179 and 0.000171. Symbols and abbreviations: Excitatory time constant, τ_{e}; Inhibitory time constant, τ_{i}; Long-range coupling constant, α; Conduction velocity, S; Excitatory gain, G_{EI}; Inhibitory gain, G_{II}.

Supplementary Figure 19: Regression Diagnostics for Age Prediction Models

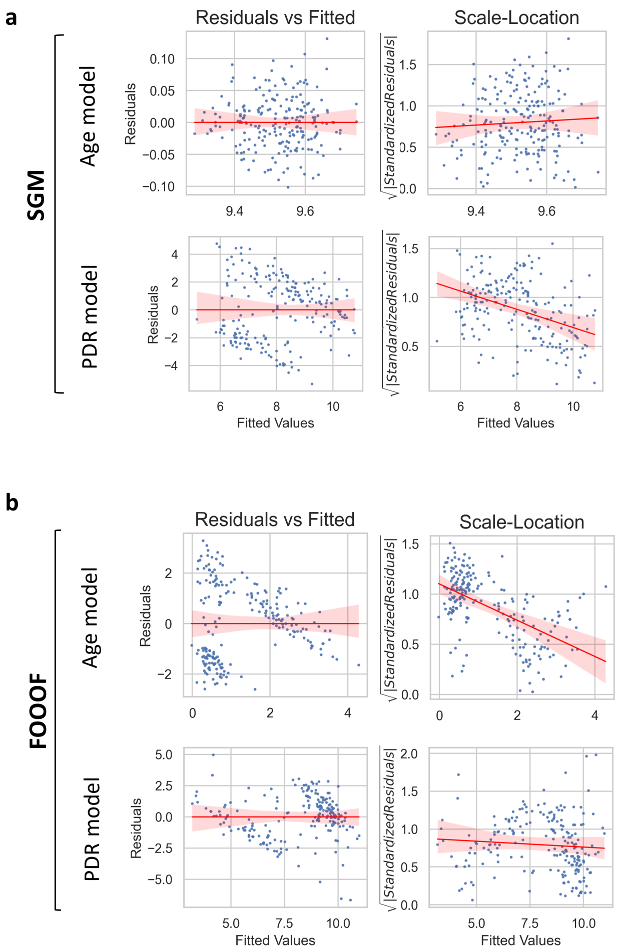

The left column demonstrates residuals vs. fitted values, and the right column demonstrates scale-location plots for the Spectral Graph Model (SGM) and Fitting Oscillations & One Over F (FOOOF) regression models for age and PDR prediction, respectively. The red line in the residuals vs. fitted plots shows the relationship between the fitted values (predicted values) and the residuals for each subject (blue dots), with the shaded regions indicating the 99.9% confidence interval. The red lines in the scale-location plots show the relationship between the fitted values (predicted values) and the square root of the absolute standardized residuals, with shaded regions indicating the 99.9% confidence intervals. Significant heteroskedasticity was found for the SGM PDR and FOOOF age regression models, with χ^2 of 20.52 and 57.4, respectively, with p-values less than 1e-3.

4 Supplementary Tables

Supplementary Table 1: Computation Time for TSNPE

Simulation Budget	Time (min)
100	94
500	407
2000	2586

Computation time (min) for Truncated Sequential Neural Posterior Estimation (TSNPE) is demonstrated at different simulation budgets, utilizing 3 sequential rounds.

5 Supplementary References

References

[1] Deistler, M., Goncalves, P.J., Macke, J.H.: Truncated proposals for scalable and hassle-free simulation-based inference. Advances in Neural Information Processing Systems **35**, 23135–23149 (2022)

[2] Greenberg, D., Nonnenmacher, M., Macke, J.: Automatic posterior transformation for likelihood-free inference. In: International Conference on Machine Learning, pp. 2404–2414 (2019). PMLR

[3] Betancourt, M.: Calibrating model-based inferences and decisions. arXiv preprint arXiv:1803.08393 (2018)
