# Supplementary material for: Simulation-based inference of developmental EEG maturation with the spectral graph model
Source: Commun Phys. 2024 Jul 31;7(1):255. doi: 10.1038/s42005-024-01748-w (PMC12310519; doi:10.1038/s42005-024-01748-w)
Supplement: Supplementary file 3 — Reporting Summary [file 42005_2024_1748_MOESM3_ESM.pdf]

nature portfolio

Corresponding author(s): Danilo Bernardo

Last updated by author(s): Jul 8, 2024

Reporting Summary

Nature Portfolio wishes to improve the reproducibility of the work that we publish. This form provides structure for consistency and transparency in reporting. For further information on Nature Portfolio policies, see our Editorial Policies and the Editorial Policy Checklist.

Statistics

For all statistical analyses, confirm that the following items are present in the figure legend, table legend, main text, or Methods section.

n/a	Confirmed	
☐	☒	The exact sample size (*n*) for each experimental group/condition, given as a discrete number and unit of measurement
☐	☒	A statement on whether measurements were taken from distinct samples or whether the same sample was measured repeatedly
☐	☒	The statistical test(s) used AND whether they are one- or two-sided<br>*Only common tests should be described solely by name; describe more complex techniques in the Methods section.*
☐	☒	A description of all covariates tested
☐	☒	A description of any assumptions or corrections, such as tests of normality and adjustment for multiple comparisons
☐	☒	A full description of the statistical parameters including central tendency (e.g. means) or other basic estimates (e.g. regression coefficient) AND variation (e.g. standard deviation) or associated estimates of uncertainty (e.g. confidence intervals)
☐	☒	For null hypothesis testing, the test statistic (e.g. F, t, r) with confidence intervals, effect sizes, degrees of freedom and P value noted<br>*Give P values as exact values whenever suitable.*
☐	☒	For Bayesian analysis, information on the choice of priors and Markov chain Monte Carlo settings
☐	☒	For hierarchical and complex designs, identification of the appropriate level for tests and full reporting of outcomes
☐	☒	Estimates of effect sizes (e.g. Cohen's *d*, Pearson's *r*), indicating how they were calculated

Our web collection on statistics for biologists contains articles on many of the points above.

Software and code

Policy information about availability of computer code

Data collection	*Provide a description of all commercial, open source and custom code used to collect the data in this study, specifying the version used OR state that no software was used.*
Data analysis	The FOOOF algorithm python package (version 1.1) was used to parameterize neural power spectra. The sbi python package (version 0.22) was used for simulation based inference. Code for the spectral graph model (SGM) can be found on the spectrome package GitHub page (https://github.com/Raj-Lab-UCSF/spectrome). Code for incorporation of the SGM with SBI are available on the project's GitHub page (https://github.com/dbernardo05/sbi-spectrome).

For manuscripts utilizing custom algorithms or software that are central to the research but not yet described in published literature, software must be made available to editors and reviewers. We strongly encourage code deposition in a community repository (e.g. GitHub). See the Nature Portfolio guidelines for submitting code & software for further information.

Data

Policy information about availability of data

All manuscripts must include a data availability statement. This statement should provide the following information, where applicable:

- Accession codes, unique identifiers, or web links for publicly available datasets
- A description of any restrictions on data availability
- For clinical datasets or third party data, please ensure that the statement adheres to our policy

EEG data were obtained from publicly available neonatal and infant EEG datasets with an age range of one month to one year of age (Xiao R, at al. PLoS One. 2018 Jan 12;13(1):e0190276.) and publicly available database of EEGs containing ages 1 to 30 years of age (Alexander LM, et al. Scientific data. 2017 Dec 19;4(1):1-26.). The power spectra derived from the publicly available EEG samples used for SBI are available from the corresponding author upon reasonable request. EEG data for subjects between one day and five years were obtained at the University of California San Francisco (UCSF) and raw patient-related data are not available due to data privacy laws. Pre-processed UCSF EEG data are available under restricted access due to ethical and privacy reasons. UCSF EEG data can be requested by contacting the corresponding author, and data sharing is conditional to the establishment of a specific data-sharing agreement between the applicant's institution and UCSF.

Research involving human participants, their data, or biological material

Policy information about studies with human participants or human data. See also policy information about sex, gender (identity/presentation), and sexual orientation and race, ethnicity and racism.

Reporting on sex and gender	No sex or gender based analyses were performed.
Reporting on race, ethnicity, or other socially relevant groupings	No analyses based on race, ethnicity, or other socially relevant groupings were performed.
Population characteristics	Ages of subjects included in the analyses were reported.
Recruitment	Subject EEG data from the datasets used from the following publically available datasets were collected after informated consent was obtained from a parent or legal guardian before participation, as described within the original study: Xiao R, at al. PLoS One. 2018 Jan 12;13(1):e0190276.; and Alexander LM, et al. Scientific data. 2017 Dec 19;4(1):1-26.<br>Subject EEG data from University of California, San Francisco (UCSF) were obtained in retrospective study following IRB approval from the UCSF IRB.
Ethics oversight	University of California, San Francisco (UCSF) IRB for UCSF EEG Data. University of Southern California for data obtained from Xiao R, at al. PLoS One. 2018 Jan 12;13(1):e0190276. Chesapeake Institutional Review Board for data obtained from Alexander LM, et al. Scientific data. 2017 Dec 19;4(1):1-26.

Note that full information on the approval of the study protocol must also be provided in the manuscript.

Field-specific reporting

Please select the one below that is the best fit for your research. If you are not sure, read the appropriate sections before making your selection.

☒ Life sciences ☐ Behavioural & social sciences ☐ Ecological, evolutionary & environmental sciences

For a reference copy of the document with all sections, see nature.com/documents/nr-reporting-summary-flat.pdf

Life sciences study design

All studies must disclose on these points even when the disclosure is negative.

Sample size	Sample size was based on number of EEG samples that were available publicly and through University of California, San Francisco (UCSF).
Data exclusions	No data were excluded.
Replication	Attempts at replication were successful.
Randomization	No randomization was required by the study design.
Blinding	No blinding was required by study design as this was a retrospective study.

Reporting for specific materials, systems and methods

We require information from authors about some types of materials, experimental systems and methods used in many studies. Here, indicate whether each material, system or method listed is relevant to your study. If you are not sure if a list item applies to your research, read the appropriate section before selecting a response.

Materials & experimental systems

n/a	Involved in the study
☒	☐ Antibodies
☒	☐ Eukaryotic cell lines
☒	☐ Palaeontology and archaeology
☒	☐ Animals and other organisms
☒	☐ Clinical data
☒	☐ Dual use research of concern
☒	☐ Plants

Methods

n/a	Involved in the study
☒	☐ ChIP-seq
☒	☐ Flow cytometry
☒	☐ MRI-based neuroimaging

Plants

Seed stocks	Not applicable.
Novel plant genotypes	Not applicable.
Authentication	Not applicable.
